# Complete Ring Artifacts Reduction Procedure for Lab-Based X-ray Nano CT Systems

**DOI:** 10.3390/s21010238

**Published:** 2021-01-01

**Authors:** Jakub Šalplachta, Tomáš Zikmund, Marek Zemek, Adam Břínek, Yoshihiro Takeda, Kazuhiko Omote, Jozef Kaiser

**Affiliations:** 1 CEITEC—Central European Institute of Technology, Brno University of Technology, Purkyňova 656/123, 612 00 Brno, Czech Republic; jakub.salplachta@ceitec.vutbr.cz (J.Š.); tomas.zikmund@ceitec.vutbr.cz (T.Z.); marek.zemek@ceitec.vutbr.cz (M.Z.); adam.brinek@ceitec.vutbr.cz (A.B.); 2 Rigaku Corporation, 3-9-12, Matsubara-cho, Akishima-shi, Tokyo 196-8666, Japan; y-takeda@rigaku.co.jp (Y.T.); omote@rigaku.co.jp (K.O.)

**Keywords:** ring artifacts reduction, CCD detector, sCMOS detector, high-resolution X-ray computed tomography, relative total variation

## Abstract

In this article, we introduce a new ring artifacts reduction procedure that combines several ideas from existing methods into one complex and robust approach with a goal to overcome their individual weaknesses and limitations. The procedure differentiates two types of ring artifacts according to their cause and character in computed tomography (CT) data. Each type is then addressed separately in the sinogram domain. The novel iterative schemes based on relative total variations (RTV) were integrated to detect the artifacts. The correction process uses the image inpainting, and the intensity deviations smoothing method. The procedure was implemented in scope of lab-based X-ray nano CT with detection systems based on charge-coupled device (CCD) and scientific complementary metal-oxide-semiconductor (sCMOS) technologies. The procedure was then further tested and optimized on the simulated data and the real CT data of selected samples with different compositions. The performance of the procedure was quantitatively evaluated in terms of the artifacts’ detection accuracy, the comparison with existing methods, and the ability to preserve spatial resolution. The results show a high efficiency of ring removal and the preservation of the original sample’s structure.

## 1. Introduction

In the field of high-resolution X-ray computed tomography (CT) with a micron and submicron spatial resolution, reconstructed CT data are often affected by severe ring artifacts. They appear as concentric ring-like features superimposed on the imaged scene and are centered on the object’s center of rotation creating either full rings (full scan over 360°) or half rings (half scan over 180°) [1]. Ring artifacts are mainly caused by imperfect detector pixels, where a perfect pixel’s response should be linearly proportional to the amount of photons incident on the detector. There are many different underlying causes for individual pixels to have imperfect responses. These include defects in the scintillator, the detector itself, and the readout electronics [2]. Moreover, the detector responses may vary due to numerous time-dependent drifts, such as thermal drifts, and also due to changes in the X-ray spectrum [2]. No matter the cause, ring artifacts degrade the resulting image quality. Therefore, it is desirable to remove or at least significantly reduce the presence of ring artifacts in CT data.

Ring artifacts reduction methods can be divided into three approaches. The first is based on a flat-field correction of a detector [3]. The proper flat-field correction should ideally remove all the detector sensitivity variations [4]. However, ring artifacts may persist after this correction due to the detector pixels intensity dependencies and non-linear response functions, or due to time-dependent non-uniformities of the incident beam [5]. To overcome these issues, advanced flat-field correction approaches were proposed in several recent works [2,4,6,7]. However, such sophisticated flat-field methods are not easily applicable in practice, because they require specific CT acquisition scenarios, and precise knowledge of used detection system is needed.

Second approach is the hardware-based ring artifacts reduction method. This method is based on moving the detector system in defined horizontal steps during the CT acquisition so that the object is projected on different regions of the detector during a CT scan [8]. Through this, the effect of non-uniform detector responses is suppressed. Although the practical functionality of this method was reported in [9] and [10], this procedure’s disadvantage is reducing the spatial resolution of the CT data if the detector shifts are not accurately known [2] or the movement precision is worse than the used detector pixel size. In general, this method is hardly applicable in nano CT systems due to such demanding requirements on the movement precision.

The third approach for the ring artifacts reduction are the image-based processing methods. These methods can be further divided, based on the domain of processed data, to sinogram-based (sinogram pre-processing) and tomogram-based (CT data post-processing) methods [11]. Sinogram-based methods work directly with the sinogram data, where the ring artifacts appear as straight lines in a vertical direction and are therefore easier to detect and to process. Some of these methods assume the presence of a specific high-frequency component that is directly related to the ring artifacts. Therefore, they aim to filter out the artifacts using low pass filters [12,13,14,15]. Most of these, however, fail to remove the strong artifacts related to dead detector elements or damaged areas on the scintillator, in which case they create an extra band around the original ring [10]. To overcome this, other methods first detect the ring artifacts elements and then correct them using various approaches: image inpainting [1,16,17,18] moving average and weighted moving average filters [19,20,21], sensitivity equalization [22]. However, even these methods have their limitations. Most of these methods are only suitable for suppressing a certain type of stripe. Moreover, they are generally difficult to use in practice due to many parameters needing to be adjusted when a wrong selection of parameters significantly affects the resulting quality. However, the work of Anas [16] can be pointed out because it introduced a novel idea for classifying rings based on their statistical properties and for addressing each type separately.

On the contrary, the tomogram post-processing methods work with CT data after the tomographic reconstruction. These methods often use a conversion of the data from Cartesian to the polar coordinate system. After this conversion, the ring artifacts appear as stripes that can be further processed using similar assumptions and strategies as for sinogram-based methods [23,24,25,26,27]. One method from these can be pointed out, Liang [26] proposed a novel ring artifacts reduction approach integrating benefits of an efficient iterative framework together with relative total variations (RTV) algorithm for the texture extraction. However, this method uses a simple mean values analysis to detect and correct the artifacts, which is insufficient in the case of dead detector elements or damaged areas on the scintillator. Moreover, tomogram-based methods are, in general, strongly dependent on the quality of the used tomographic reconstruction because some extra artifacts might be created [20]. Therefore, a novel class of methods lying between sinogram-based and tomogram-based approaches has been recently developed. The ring artifacts reduction is addressed directly during the reconstruction process using specific forms of regularizations (e.g., [11,28,29]). Such regularizations can, however, be highly computationally demanding, which limits the practical applicability of those methods.

In this article, we present a new ring artifacts reduction procedure that combines several selected ideas from image-based processing methods into one complex sinogram-based method with a goal to overcome all previously mentioned limitations. The ring artifacts are classified into two types based on their cause and actual appearance in the CT data. We prefer to separate the detection and correction schemes for each type of artifact for their effective removal. We propose a two-step iterative correction scheme that deals with all the artifact types in the sinogram domain. Consequently, a significant influence of tomographic reconstruction on the efficiency of artifacts reduction is avoided. The reduction strategy was optimized for each artifact type separately to preserve the spatial resolution and sample’s structural information, which are the most important factors in the field of nano-tomography. Practical functionality of the prosed method was verified on both synthetic data and real CT data. It shows a high efficiency of ring artifacts removal, and a robustness to character of input data and used detection system in context of other tested ring artifacts correction techniques.

## 2. Materials and Methods

**Figure 1:**
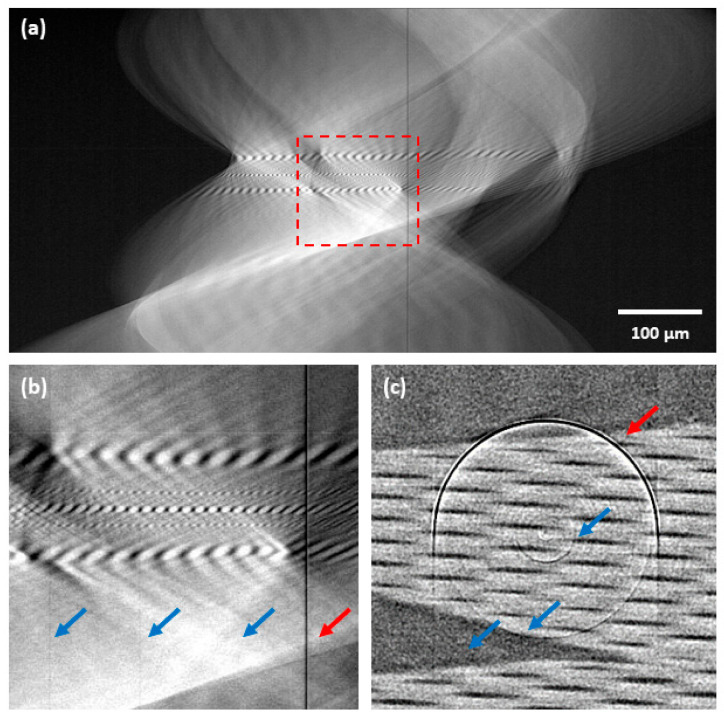
Example of ring artifacts affected data—glass capillary sample acquired with a charge-coupled device (CCD)-based camera: (**a**) sinogram; (**b**) detail of sinogram central area; (**c**) central area of corresponding tomogram. Red arrows indicate the high-level ring artifacts and blue arrows the low-level ring artifacts.

In this article, a two-step ring artifacts reduction scheme is proposed. This scheme was developed for the artifacts’ reduction in the sinogram domain and is based on a categorization of ring artifacts into two types. The ring artifacts are categorized based on the observation of responses from different kinds of deficiencies in sinograms and on their specific hardware causes (see Figure 1). In the proposed reduction scheme, each of these classes is then addressed separately using dedicated detection and reduction procedures. In the first step, the most prominent ring artifacts (high-level artifacts) are corrected, and subsequently weak artifacts (low-level artifacts) are corrected in the second step.

### 2.1. High-Level Ring Artifacts

The class of high-level ring artifacts (HRA) is represented by the most prominent stripes in the sinograms (see Figure 1). The actual cause of such artifacts is two-fold. One cause originates from entirely dead detector pixels or damaged areas on the scintillator. The behavior of these pixels then does not follow the pattern of responses of adjacent non-defective elements. Their responses are close or equal to the saturation level of the detector (maximum of the dynamic range) or the minimum of the dynamic range. The second cause is related to so-called “hot pixels”, which may be considered as a type of fixed pattern noise [30]. They are defined as pixels with the dark current values significantly above the average. They follow the responses of the adjacent non-defective pixels but with significant deviations that do not vary in time. In the sinograms, they appear as prominent stripes, but they do not reach the extremes of the dynamic range.

#### High-Level Ring Artifacts Removal

**Figure 2:**
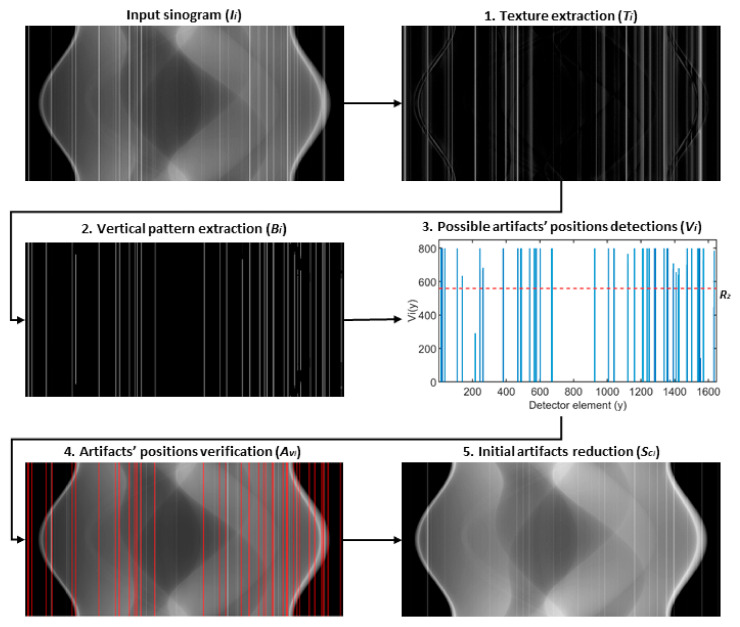
Illustrative scheme of proposed high-level ring artifacts detection scheme—as example images, the outputs from the first iteration are shown.

The reduction in high-level ring artifacts is divided into two parts: first, the artifacts’ positions are detected, and second, the input sinogram is corrected at these positions. For the artifacts’ detection, an iterative detection scheme was designed. This detection procedure consists of 5 steps (see Figure 2) that are iteratively repeated until any of 4 stopping conditions is fulfilled. These steps are:

1. Texture extraction

Ring artifacts, together with the structural details, are considered as a texture of sinogram. Therefore, textural information is first extracted using the subtraction of input sinogram and its smoothened version (i.e., image after texture removal):

(1) $$T_{i}=I_{i}-\;S_{s_{i}},$$

where *T_i_* is extracted texture image in the current iteration *i*, *I_i_* is input sinogram in the current iteration *i* and *S_s,i_* is input sinogram after the texture removal. For texture removal, an algorithm based on relative total variation (RTV) is used (for more details see the Appendix A section). After the texture extraction in the current iteration, the first stopping condition (SC 1) is evaluated:

(2) $$\frac{\|T_{i}-\;T_{i-1}\|{}_{2}}{\|T_{1}\|{}_{2}}\;\leq R_{1},$$

where *T_i_* is extracted texture image in the current iteration *i*, *T_i−_*_1_ is extracted texture image in the previous iteration, *T*_1_ is extracted texture image in the first iteration, and *R*_1_ is a selected threshold value. Using this stopping condition, the iteration is stopped when the normalized L2 norm of the difference between two sequential extracted textures is equal or lower than the set value *R*_1_.

2. Vertical pattern extraction

The extracted texture image from the previous step is further convolved:

(3) $$P_{i}=\;T_{i}\;*\;k,$$

with a convolution kernel *k* that corresponds to one-dimensional (1D) vertical mean filter with the length *l*. This is done to highlight a vertical stripe pattern (e.g., ring artifacts) and to blur remaining non-vertical structures. Then, the first derivative is approximated by finite differences in the horizontal direction, and the result is binarized row-by-row by thresholding with a threshold value set to a double of the calculated standard deviation of a given row:

(4) $$B_{i}\left(x,y\right)=\left\{\begin{array}{ll} 1, & if\;{∆}_{x}P_{i}{}_{x}\left(y\right)>2\;\cdot \;\sigma_{{∆}_{x}P_{i}{}_{x}}\\ 0, & otherwise \end{array}\right.,$$

where *B_i(x,y)_* corresponds to the value of resulting binary mask in the current iteration *i* at coordinates *x*,*y* and *σ* is used notation for standard deviation.

3. Possible artifacts’ positions detections

The binary mask is then summed in the column direction:

(5) $$V_{i}\left(y\right)\;=\;\overset{M}{\underset{x=1}{\sum }}B_{i}\left(x,y\right),$$

where *V_i(y)_* refers to value of the resulting vector at position *y* and *M* refers to number of rows in the binary mask *B_i_*, *x* and *y* refer to vertical and horizontal indices, respectively. In the resulting vector, only elements with values above threshold *R*_2_ are considered as possible candidates for positions of ring artifacts *A_p_*:

(6) $$A_{p}{}_{i}\left(y\right)\;=\;\left\{\begin{array}{ll} 1, & if\;V_{i}\left(y\right)>R_{2}.\\ 0, & otherwise \end{array}\right.$$

However, the inevitable RTV smoothing errors may negatively affect this detection. To avoid this, the distances between possible detected artifacts are also analyzed. When the distance between two neighboring possible artifacts’ positions is below threshold *R*_3_, the intermediate positions are also considered as the possible artifacts’ positions. In this step, the second stopping condition (SC 2) is evaluated, the iteration is stopped when no possible artifacts’ positions are detected.

4. Artifacts’ positions verification

Verification of detected possible artifacts’ positions *A_p_* is achieved by the analysis of mean column vector *L_Ti_* of extracted textural information *T_i_* in the current iteration *i*. Possible artifact positions *A_p_* are considered as verified *A_v_*, if they meet the following condition:

(7) $$A_{v}{}_{i}\left(y\right)=\left\{\begin{array}{ll} 1, & if\;\left|L_{T_{i}}\left(A_{p}{}_{i}\left(y\right)\right)-L_{T_{i}}\left(A_{p}{}_{i}\left(y_{x}\right)\right)\right|>2\cdot \sigma_{∆L_{T_{i}}},\\ 0, & otherwise \end{array}\right.$$

where *A_p__i_(y_x_)* is the nearest artifact-free position to analyzed possible artifacts’ position *A_p__i_(y)* and the threshold value corresponds to twice the standard deviation value of the first derivative of *L_Ti_*, which is approximated by finite differences. If no possible artifacts’ positions are considered as verified, the iteration is stopped (the third stopping condition—SC 3). On the other hand, when certain positions are verified in the current iteration *i*, they are then compared to the verified positions from previous iterations and if no new verified artifacts’ position is detected, the iteration is stopped (the fourth stopping condition—SC 4).

5. Initial artifacts reduction

In each iteration, the sinogram is corrected at new verified artifacts’ positions. This is achieved by filling the sinogram at artifacts’ positions by means of image inpainting. In our work, this is completed by using a partial differential equation (PDE)-based approach, where Laplace equation is solved with the Dirichlet boundary condition:

(8) $$S_{C_{i}}=\left\{\begin{array}{ll} \nabla^{2}u=0, & for\;A_{v}{}_{i}\\ u\left(x,y\right)=I_{i}\;\left(x,y\right), & on\;\partial A_{v}{}_{i} \end{array},\right.$$

where *Sc* corresponds to corrected sinogram in the current iteration *i*.

After the termination of the detection procedure, the final artifacts reduction is conducted. In this step, the previously described inpainting scheme is used again, and the input sinogram is corrected at the detected ring artifacts’ positions *A_v_*. The implementation details and used parameters can be found in Appendix C (Table A3).

### 2.2. Low-Level Ring Artifacts

Low-level ring artifacts (LRA) are caused by miscalibrated detector pixels. Their sensitivity deviations result from higher or lower dark current values compared to the non-defected pixels. They follow the responses of the adjacent non-defective pixels but with certain deviations. In the sinogram (see Figure 1), their presence is not distinct from the non-defective pixels as the HRA, but they still negatively affect the data quality.

#### Low-Level Ring Artifacts Removal

**Figure 3:**
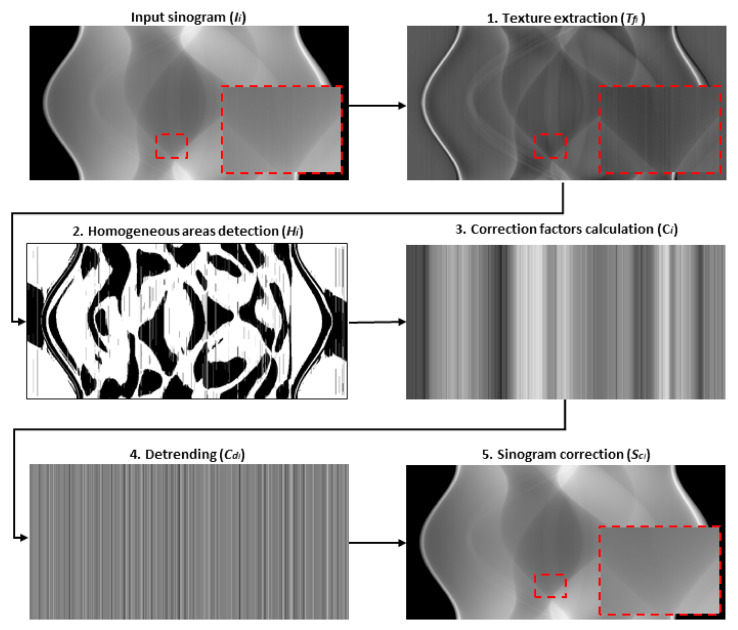
Illustrative scheme of the proposed low-level ring artifacts removal procedure—the outputs from the first iteration are shown as examples.

The main idea of the proposed algorithm for LRA removal is that column-wise neighboring homogenous areas (i.e., areas at same vertical positions of two adjacent columns) from extracted texture should ideally (without any artifact) have the same average values. To achieve this, an iterative procedure was proposed (see Figure 3), consisting of these steps:

1. Texture extraction

For the texture extraction, the same procedure as in high-level ring artifacts removal (Equation (1)) is used with the same stopping condition (Equation (2)) set to the threshold value *R*_1_. To reduce the presence of noise and its negative effect on the subsequent analysis, the extracted texture is further filtered with a 1D pixel-wise adaptive low-pass Wiener filter in column-wise direction:

(9) $$T_{f_{i}}=\;T_{i}\;*w,$$

where *T_i_* is extracted texture in current iteration *i*, *w* is kernel of 1D Wiener filter with the length *l* and *T_fi_* is the noise reduced texture, which is used only within steps 2 and 3.

2. Homogenous areas detection

Homogenous texture areas are detected column-wise using the following formula:

(10) $$H_{i}\left(x,y\right)=\left\{\begin{array}{ll} 1, & if\;\;T_{f_{i}}\left(x,y\right)\leq \bar{\;T_{f}{}_{i}\left(1:M,y\right)}\\ 0, & otherwise \end{array},\right.\;$$

where *y* is the coordinate of analyzed column, *H_i_(x,y)* corresponds to the value of resulting binary mask in the current iteration *i* at coordinates *x*,*y* and *M* is the number of sinogram rows.

3. Correction factors calculation

Correction factors for sinogram columns are calculated from extracted texture *T_fi_* by comparing two neighboring columns in terms of average intensity values of their neighboring homogenous areas. The column with smaller index is always taken as a reference, and other column is then corrected using the following equation:

(11) $$\;T_{f_{i}}\left(1:M,y\right)=T_{f_{i}}\left(1:M,y\right)+C_{i}\left(y\right),$$

where *C_i_(y)* is a correction factor for column *y* in the current iteration *i*:

(12) $$C_{i}\left(y\right)=\left(\frac{\sum_{j=1}^{N_{h}}\;T_{f_{i}}\left(h,y-1\right)}{N_{h}}-\frac{\sum_{j=1}^{N_{h}}\;T_{f_{i}}\left(h,y\right)}{N_{h}}\right),$$

where *h* is *x* coordinates of neighboring homogenous areas in analyzed columns (at coordinates *y* and *y*−1), *h* is those coordinates where $H_{y}=1\;\wedge$ $H_{y-1}=1$, and *N_h_* is the number of those positions. If *N_h_* = 0, the correction factor for the previous column (C*_y_*_−1_) is used.

4. Detrending

Calculated correction factors in the previous step can successfully reduce the artifacts, but the overall structural trend of the extracted texture is also lost. To avoid this, the Savitzky-Golay filter [31] is used to extract this trend from the calculated correction factor values:

(13) $$t=\;C_{i}\;*s,\;$$

where *s* is the 1D kernel of the Savitzky-Golay filter with polynomial order *r* and frame length *f*. The value of this parameter *f* is decreasing with each iteration by power of two—by this the over-smoothing effect is avoided. Subsequently, this trend is subtracted from the calculated correction factors:

(14) $$C_{d_{i}}=\;C_{i}-t,$$

to ensure that only the artifacts are reduced while the overall structural pattern of the textural information is preserved.

5. Sinogram correction

In each iteration *i*, the corrected sinogram *S_C_* is calculated as the sum of the smoothened sinogram *S_s_* (i.e., sinogram after texture removal) and the extracted texture after an artifact reduction:

(15) $$S_{C_{i}}=\;S_{s_{i}}+\left(T_{i}+C_{d_{i}}\right).$$

These steps are repeated until the stopping condition described by Equation (2) is fulfilled. The implementation details and used parameters can be found in Appendix C (Table A4).

### 2.3. Real CT Data Acquisition

A laboratory-based CT system Rigaku nano3DX [32] was used for CT measurements. For purposes of our study, this CT system was equipped with a Rigaku’s scientific X-ray CDD camera (XSight™ Micron LC X-ray CCD camera [33]) and a scientific X-ray scientific complementary metal-oxide-semiconductor (sCMOS) camera (XSight™ Micron LC X-ray sCMOS camera [34]), nominal parameters of used cameras are stated in Table A2 in Appendix B. As it was shown in our previous study [35], radiographic data acquired by tested charge-coupled device (CCD) and sCMOS cameras mainly differ in projection domain in terms of the population of hot pixels that mostly correspond to high-level ring artifacts. As samples, a glass capillary array (pores diameter: 3 μm) and a ruby ball (diameter: 300 μm) were selected. They were scanned using circular trajectory with an angular range from 0 to 180 degrees with an acquisition of 800 projection images for one CT scan. Molybdenum rotatory target was used (50 kV and 24 mA) for all the measurements. Exposure times for X-ray projection data were selected following the manufacturer’s recommendations (based on the level of detected signal). Specifically, the exposure times for glass capillary array measurements were 16 s (CCD) and 6 s (sCMOS), and they were 13 s (CCD) and 4.5 s (sCMOS) for ruby ball measurements. Acquired projection data were only flat-field corrected before the ring artifacts reduction was applied. Subsequently, CT data were reconstructed using ASTRA toolbox [36]—filtered back projection (FBP) reconstruction with cosine filter. Then, all the data were normalized so that the minimum and maximum values were 0 and 1 arbitrary units, respectively. The achieved linear voxel size values for binning 2 × 2 were 0.53 µm and 0.63 µm for the CCD and sCMOS cameras, respectively.

### 2.4. Synthetic Data Creation

**Figure 4:**
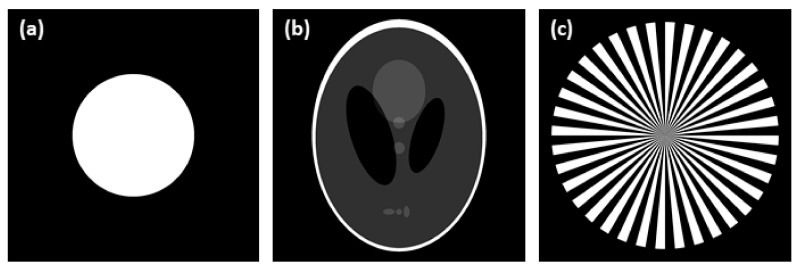
Synthetic data used for testing and validation of all tested methods: (**a**) ball phantom; (**b**) Shepp-Logan phantom; (**c**) Siemens star phantom.

Three synthetic images were used in this work, representing various levels of data complexity—a ball phantom (single material sample), a Shepp-Logan phantom (multi material sample) and a Siemens star phantom (highly complex sample). Phantom images were generated in tomogram domain (see Figure 4) and then transformed by Radon transform to sinogram domain, using the ASTRA Tomography Toolbox [36]. The sinograms were simulated to have similar parameters as those acquired by nano3DX device equipped with a CCD camera, specifically to have a linear voxel size of 0.53 µm, a detector width of 1648 pixels and to follow the acquisition of 800 projection angles from an angular range of 0° to 180°. Gaussian distributed noise with a standard deviation of 0.01 (reflects noise properties of real projection data) was also added to generated sinograms.

**Figure 5:**
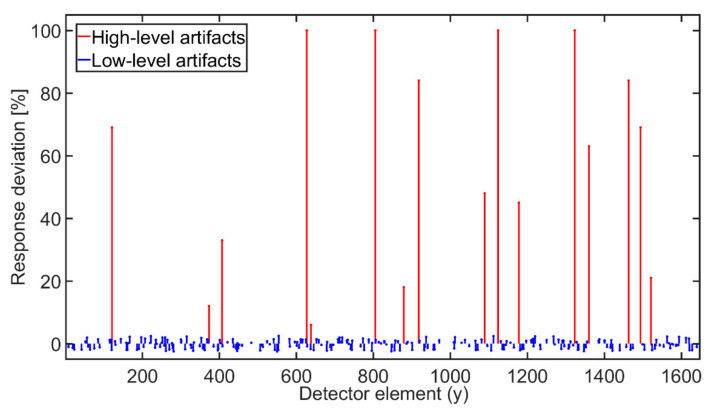
Example of simulated response deviations of detector elements representing the ring artifacts.

**Figure 6:**
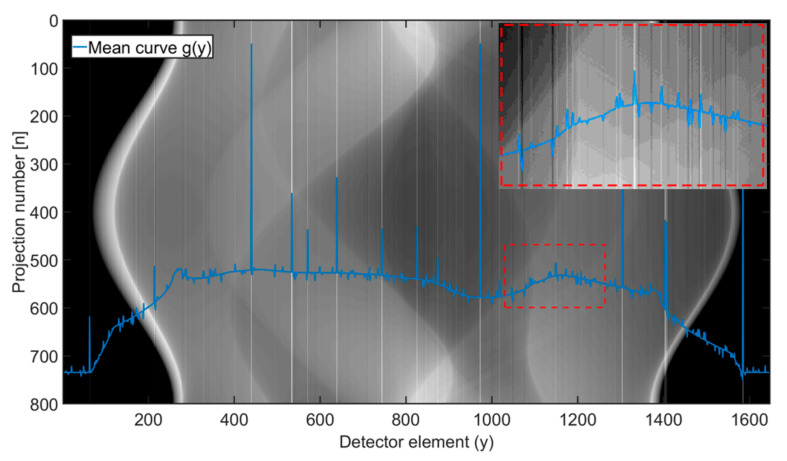
Shepp-Logan phantom sinogram affected by simulated ring artifacts presented in Figure 5. Blue curve shows mean column values—the highest peaks belong to high-level ring artifacts (HRA). In the red labelled image, the magnified area affected by low-level ring artifacts (LRA) is shown.

The ring artifacts were simulated (see Figure 5) and added to the sinograms (see Figure 6). In total, 25% of detector elements were affected by artifacts: 5% HRA and 20% LRA. The artifacts’ positions were generated randomly without any recurrences. As for high-level artifacts, one fifth of affected positions was assigned the intensity value (referring to detector response) equal to the maximum of used dynamic range (16bit), which corresponds to dead, unresponsive detector pixels. The remaining high-level artifacts’ positions were assigned the intensity deviations generated as uniformly distributed random numbers from the interval from 10% to 60% of maximum sinogram intensity value. The intensity deviations of low-level artifacts were generated similarly but from the interval ± 1% of maximum sinogram intensity value. Such deviations were then added to the original responses at given artifacts’ positions. Using such parameters, an extreme case of ring artifacts presence in sinogram was simulated.

### 2.5. Other Methods

The performance of the proposed method was compared to three other selected approaches from the class of sinogram-based methods: the wavelet-Fourier-transform-based method by Münch [13], correction vector-based method by Eldib [15] and complex correction technique by Vo [21]. For notational simplicity, these methods are further called M1, M2 and M3, respectively. Since the performance of all the methods is highly dependent on specific settings, the optimal parameters for each method were selected to ensure a relevant and fair comparison. This was completed based on the suggestions in the original works and also based on the practical testing on synthetic data using a combination of both qualitative and quantitative evaluation (brute-force search with structural similarity index (SSIM) [37] as a validation metric). To test the consistency of these parameters within various test samples, only one specific setting was used for each of the methods (see Table A5).

### 2.6. Evaluated Criteria and Metrics

The proposed method was tested on both synthetic and real high-resolution CT data. Three criteria were considered for the evaluation. First, the proposed method was tested in terms of artifacts detection accuracy focused on the HRA detection. For this evaluation, three statistical metrics were used: true positive rate (TPR—ratio of correctly detected artifacts’ positions to all positions labelled as artifacts) [38], positive predictive value—precision (PPV—percentage of artifacts’ positions that were correctly detected) [39] and Dice similarity coefficient (DSC) [40].

Then, the overall performance of the proposed method was evaluated in context of other ring artifacts correction methods. This was completed both quantitatively using synthetic data with ground truth images and qualitatively on real CT data. For the quantitative performance evaluation, two metrics were used: the peak signal-to-noise ratio (PSNR), and structural similarity index (SSIM) [37]. These were calculated between the corrected tomogram (tomogram reconstructed from the ring artifacts corrected sinogram) and the ground truth tomogram (tomogram reconstructed from the corresponding sinogram without ring artifacts). The resulting tomogram data were first standardized to Z-scores, i.e., mean value was subtracted from the data and the result was divided by the corresponding standard deviation. This was completed so that the possible effect of intensity shifts on corrected data could be eliminated. It was possible to precisely evaluate the functionality of ring artifacts reduction and also the effect of distortion on the data.

The lastly considered criterion focused on the robustness of the proposed ring artifacts reduction procedure to the used detection system, and on its effect on achieved spatial resolution. For the spatial resolution calculation, the modulation transfer function (MTF) analysis [41] was used following the procedure defined in ASTM E1695-95(2013) standard [42]. CT data of the ruby ball sample, acquired by both CCD- and sCMOS- based cameras, were used for this analysis.

## 3. Results

### 3.1. HRA Detection Accuracy of Proposed HRA Detection Scheme

The accuracy of the proposed HRA detection scheme was evaluated on the synthetic data with known artifacts’ positions. The results are stated in Table 1. For all the phantom images, the proposed method was able to classify all the artifacts’ positions with a precision above 95%. However, a certain amount of artifacts’ positions, out of total 82 artifacts’ positions, was not detected in all the cases: two artifacts’ positions for Shepp-Logan and Siemens star phantoms, and three positions in the case of ball phantom. For Siemens star phantom, a higher number of falsely classified artifacts’ positions led to a PPV score of 84.21% and DSC of 90.40%. On the contrary, for Shepp-Logan phantom, all the positions classified as artifacts were correct (PPV = 100%).

**Figure 7:**
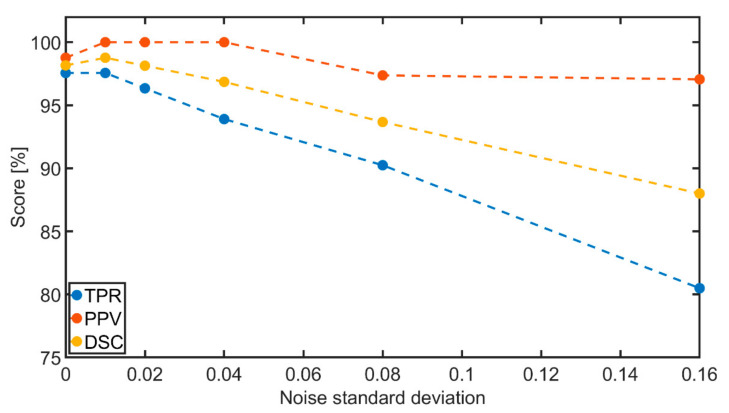
Dependence of the accuracy of the proposed HRA detection scheme on the noise level—evaluated for Shepp-Logan phantom.

We further evaluated the effect of noise level in the data on the detection accuracy of proposed HRA detection scheme (tested on Shepp-Logan phantom). Results of this analysis are shown in Figure 7. The precision of artifacts’ detection was found almost independent of the noise presence, reaching values above 95% for all tested cases. However, a direct proportion was found between the noise level and the number of artifacts’ positions that were not detected. This tendency is expressed by both the TPR and DSC metrics. Despite this tendency, the proposed HRA detection scheme resulted in scores of both metrics above 80% even for cases with a severe noise presence.

### 3.2. Overall Performance Evaluation in Context of Other Tested Methods

**Figure 8:**
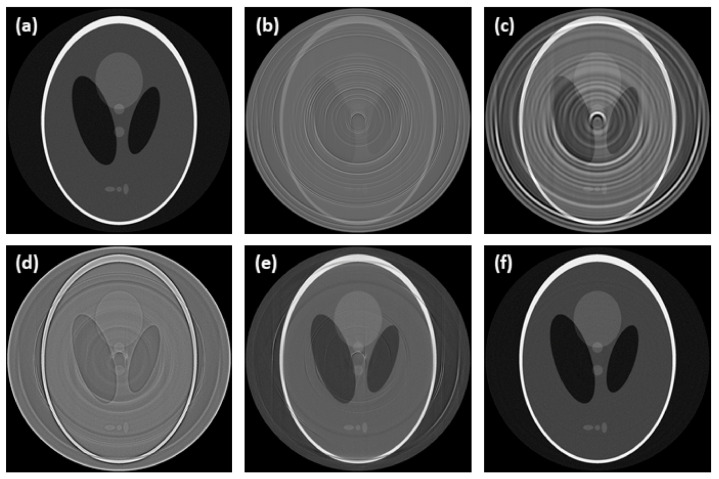
Comparison of tomograms after ring artifacts reduction by tested methods—simulated data of Shepp-Logan phantom: (**a**) reference; (**b**) original (without any correction); (**c**) M1; (**d**) M2; (**e**) M3; (**f**) proposed. For visualization, the same contrast setting was used for all the images.

**Figure 9:**
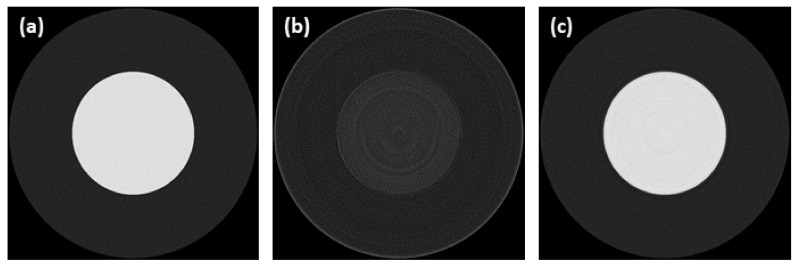
Comparison of data distortion between the proposed method and M2 method—tomograms of ball phantom: (**a**) reference; (**b**) M2 method; (**c**) proposed. For visualization, the same contrast setting was used for all the images.

**Figure 10:**
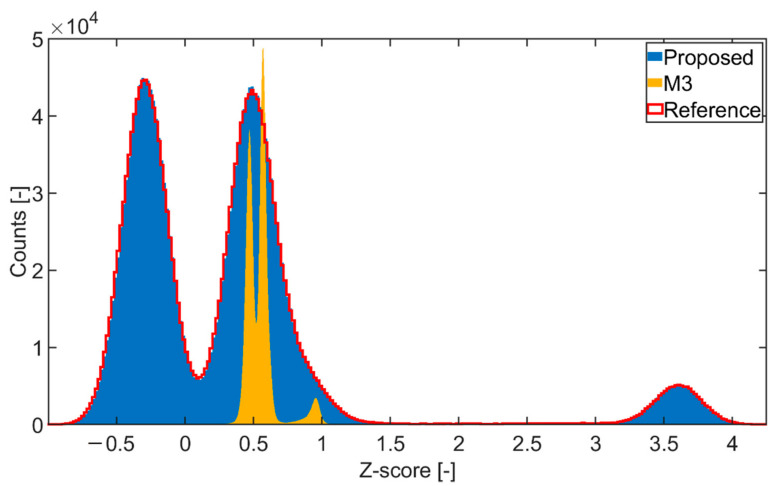
Comparison of data distortion between the proposed method and M3 method—histogram analysis of standardized tomogram values.

A high ring artifacts presence in the synthetic data made their correction very challenging, which is reflected by the poor results of the tested methods (Table 2). Apart from the proposed method, all the tested methods failed to successfully reduce the artifacts, especially the population of HRA, and to preserve the structural information (see Figure 8). The overall worst results were achieved by the M2 method. Especially in case of the ball phantom, the M2 method failed to distinguish the artifacts and sample structure. It led to an almost complete suppression of structural information (see Figure 9), which is further represented by a negative SSIM value (Table 2). In the case of the M1 method, a poor correction led to wide rings and blurring the overall image structures (see Figure 8). The M3 method results were visually good, and most of the artifacts were successfully reduced (see Figure 8). However, the quantitative evaluation (Table 2) revealed a poor input data preservation in terms of structural information and intensity values. This effect is further demonstrated in Figure 10 by histogram analysis of the Shepp-Logan phantom tomogram. Unlike the proposed method, the M3 method led to a significant transformation of histogram shape and Z-score range compared to the reference data. Compared to all other methods, the proposed method obtained the best results, as all the artifacts were reduced, and the sample structure was fully preserved (see Figure 8, Figure 9 and Figure 10).

**Figure 11:**
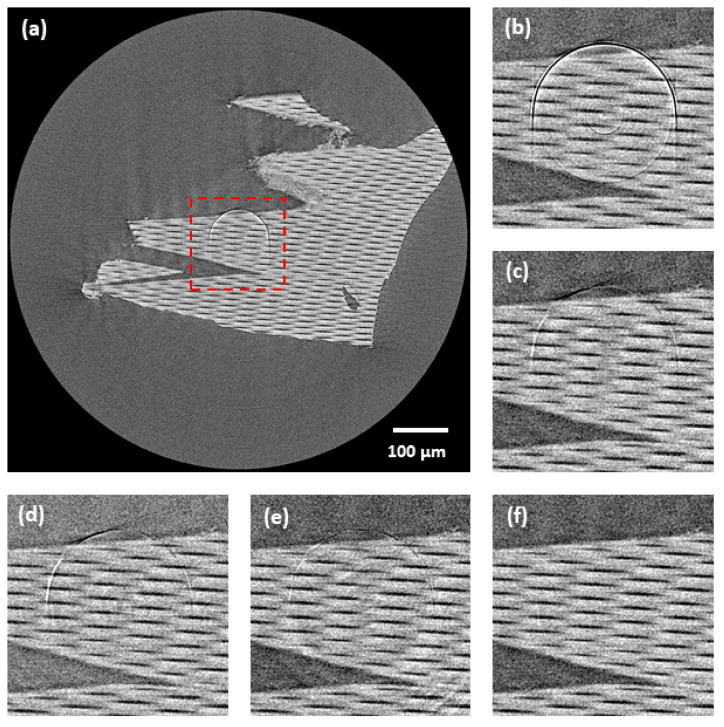
Comparison of tomograms after ring artifacts reduction by tested methods—real computed tomography (CT) data of glass capillary array acquired with a CCD-based camera: (**a**) original tomogram (without any correction)—red labelled area marks central area visualized in following images; (**b**) original; (**c**) M1; (**d**) M2; (**e**) M3; (**f**) proposed. For visualization, the same contrast setting was used for all the images.

The overall performance of the tested methods was further evaluated on real CT data. From the resulting tomograms, the effectiveness of the tested methods was evaluated qualitatively using visual perception. For visualization purposes, the glass capillary array data acquired by a CCD camera were selected due to the presence of prominent HRA in the central area (see Figure 11). Apart from the proposed method, the other methods only reduced the HRA to a certain degree, leaving the artifacts still detectable after the correction. Moreover, in the case of the M3, some extra artifacts were created during the correction (see Figure 11e).

### 3.3. Spatial Resolution Preservation and Robustness to Used Detection System

**Figure 12:**
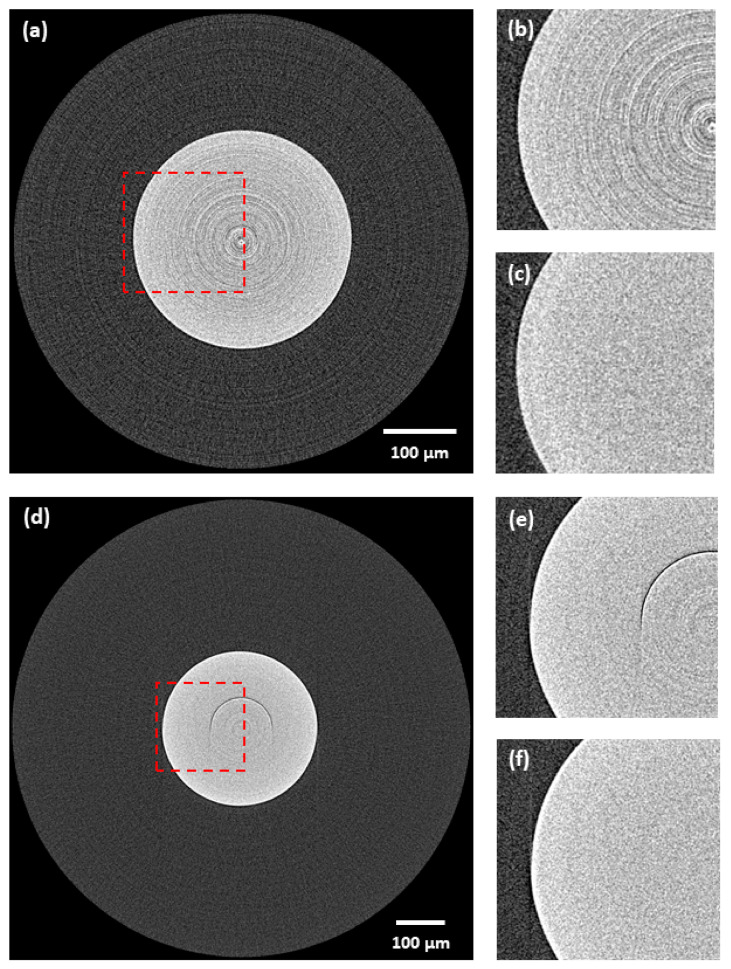
Demonstration of robustness of the proposed method to the used detection system—real CT data of ruby ball: (**a**) original tomogram (scientific complementary metal-oxide-semiconductor (sCMOS), without any correction)—red labelled area marks the central area visualized in following images; (**b**) detail (sCMOS)—original; (**c**) detail (sCMOS)—corrected by proposed method; (**d**) original tomogram (CCD, without any correction)—red labelled area marks the central area visualized in following images; (**e**) detail (CCD)—original; (**f**) detail (CCD)—corrected by proposed method. For visualization, the same contrast setting was used for all the images.

The results of the spatial resolution evaluation are stated in Table 3. The proposed method was able to preserve the spatial resolution within the accuracy limit of the used standard for both used detection systems. The robustness is further visually demonstrated in Figure 12. The proposed method in this example reduced all the ring artifacts without any negative effect or distortion on the data regardless of the used detection system.

## 4. Discussion

The practical testing demonstrated that the proposed ring artifacts reduction procedure, compared to other methods, can achieve superior results in the following criteria: artifacts detection accuracy, overall performance, robustness to detection system, and the ability to preserve the spatial resolution. First, the method was tested in terms of HRA detection accuracy. It was found that for all the tested data, the proposed HRA detection scheme achieved a precision higher than 95% (see Table 1), even for the increasing noise level present in the data (see Figure 7). However, for all data, a certain amount of artifacts’ positions was not detected. Moreover, a direct proportion was found between the number of HRA positions that were not detected and the noise level. However, this amount was found to be negligible in terms of the total number of artifacts’ positions, as both the TPR and DSC metrics scored above 80% even for cases with a severe noise presence.

Although the proposed method did not detect all the HRA positions, the overall quantitative and qualitative results were superior to other tested methods. This was achieved by the proposed two-step correction scheme, when the HRA reduction algorithm and LRA reduction algorithm are working in tandem reducing all the artifacts effectively. A high ring artifacts presence in the case of the synthetic data made their correction very challenging, which was reflected by poor results of the M1, M2 and M3 methods (see Table 2). As for the M1 method, residual rings persisted after the correction for all the tested data resulting in unusable data for further analysis. However, the worst overall results were achieved with the M2 method, as it failed to reduce all the artifacts and preserve the structural information of the input data. Specifically, in the case of ball phantom, the method failed to distinguish the artifacts and sample structure leading to an almost complete suppression of structural information (see Figure 9), which is further represented by a negative SSIM value (Table 2). The M3 method achieved visually acceptable results, but the quantitative evaluation on synthetic data revealed that the method had led to a poor preservation of structural information and intensity range of the input data (see Figure 10). All these findings were further confirmed by testing on the real CT data. The acquired results corresponded to those from testing on the synthetic data. Compared to all other methods, the proposed method obtained the best results, as all the artifacts were reduced, and the sample structures were fully preserved.

The lastly considered criterion focused on the robustness of the proposed ring artifacts reduction procedure to the used detection system and its effect on the spatial resolution. In this analysis, the proposed method proved itself to preserve the spatial resolution within the accuracy limit of the regular standard for both detection systems (see Table 3). Moreover, the proposed method was functional regardless of the detection system without the need for any settings’ optimization.

All the beforehand discussed aspects restrict the application of the M1, M2 and M3 methods in nano-tomography, where preservation of quality and structural information of input data are of key importance. Moreover, these methods were found highly dependent on the used parameters and the character of input data. Even following the original authors’ recommendations and optimization, the methods did not achieve acceptable results with one setting for all the tested data. This showed a limited robustness and applicability of these methods in practice. Only the proposed method achieved acceptable results for all the conducted tests and showed a high robustness to the character of input data in terms of structure complexity and also the used detection system.

## 5. Conclusions

The small size of detector pixels used in nano CT devices does not enable an application of any hardware-based method for removing ring artifacts, leaving image-based processing methods as the most promising way for an effective ring artifacts removal. Several approaches from this class exist but each with some deficiencies, such as the degradation of data quality and spatial resolution, which is inconsistent with the core purpose of nano-tomography. The procedure presented in this paper is based on a smart implementation of several ideas from existing methods and utilization of their advantages.

The ring artifacts are classified into two types based on their cause and actual appearance in the CT data. Each artifact class is then handled separately since it is impossible for a single approach to remove all of them. In our procedure, we applied novel iterative RTV-based algorithms in the sinogram domain to avoid any negative influence of tomographic reconstruction. The proposed procedure was optimized and tested on different types of data, cameras, and samples as well.

In confrontation with other advanced ring artifacts reduction methods, it proved its supremacy during practical tests, being robust regarding the character of input data and used detection system. Moreover, the method was able to fully preserve the input data, structural information and spatial resolution. Such features show a high potential of the proposed procedure for practical use in the field of synchrotron- or lab-based nano CT systems.

## Figures and Tables

**Table 1:** Proposed HRA detection scheme accuracy—evaluated for synthetic data.

	Ball	Shepp-Logan	Siemens Star
TPR [%]	96.34	97.56	97.56
PPV [%]	96.34	100.0	84.21
DSC [%]	96.34	98.77	90.40

**Table 2:** Quantitative performance evaluation of tested methods.

	Ball		Shepp-Logan		Siemens-Star	
	PSNR [dB]	SSIM	PSNR [dB]	SSIM	PSNR [dB]	SSIM
M1	7.59	0.39	3.95	0.29	3.66	0.22
M2	1.03	−0.03	1.81	0.27	3.21	0.21
M3	11.39	0.47	1.91	0.22	3.30	0.20
Proposed	27.48	0.97	28.45	0.97	11.17	0.72

**Table 3:** Results of spatial resolution evaluation.

	CCD	sCMOS
Original	0.62 µm ± 0.03 µm	0.85 µm ± 0.04 µm
Proposed	0.62 µm ± 0.03 µm	0.82 µm ± 0.04 µm

## Data Availability

The data and the code used for the manuscript are available for researchers on request from the corresponding author.

## Appendix A

In this work, a relative total variation (RTV)-based algorithm proposed by Xu et al. [43] is used for the texture extraction. Objective function of this algorithm can be expressed as:

(A1) $$\arg\underset{S}{\min}\underset{p}{\sum }{\left(S_{p}-I_{p}\right)}^{2}+\lambda \cdot \left(\frac{D_{x}\left(p\right)}{L_{x}\left(p\right)+\varepsilon }\;+\frac{D_{y}\left(p\right)}{L_{y}\left(p\right)+\varepsilon }\right).$$

The data term ${\left(S_{p}-I_{p}\right)}^{2}$ makes the input *I* and result *S* not extensively deviate, where *p* corresponds to pixel indices. The second part of the objective function, $\lambda \cdot \left(\left(Dx\;\left(p\right)\right)/\left(Lx\;\left(p\right)+\varepsilon \right)+\left(Dy\;\left(p\right)\right)/\left(Ly\;\left(p\right)+\varepsilon \right)\right)$, corresponds to the RTV measure, where *λ* is a weight controlling the degree of smoothing, and *ε* is a small positive number to avoid division by zero and also controlling the sharpness of the image *S*. *D_x_(p)* and *D_y_(p)* are windowed total variations in the *x* and *y* directions for pixel *p*:

(A2) $$D_{x}\left(p\right)=\;\underset{q\in R\left(p\right)}{\sum }g_{p,q}\cdot \left|{\left(\partial_{x}S\right)}_{q}\right|,\;$$

(A3) $$D_{y}\left(p\right)=\;\underset{q\in R\left(p\right)}{\sum }g_{p,q}\cdot \left|{\left(\partial_{y}S\right)}_{q}\right|,\;$$

which count the absolute spatial difference within the rectangular window *R(p)* centered at pixel *p.* Pixel *q* belongs to *R(p)*. The term $g_{p,q}\;$ refers to a weighting function defined according to spatial affinity:

(A4) $$g_{p,q}\propto \exp\left(-\frac{{\left(x_{p}-x_{q}\right)}^{2}+{\left(y_{p}-y_{q}\right)}^{2}}{2\sigma^{2}}\right),$$

where *σ* controls the spatial scale of the window corresponding to the size of textural elements. To help distinguish the prominent structures from the texture elements, the RTV measure also contains windowed inherent variations *L_x_(p)* and *L_y_(p)* in the directions x and y, defined as:

(A5) $$L_{x}\left(p\right)=\;\left|\underset{q\in R\left(p\right)}{\sum }g_{p,q}\cdot {\left(\partial_{x}S\right)}_{q}\right|,\;$$

(A6) $$L_{y}\left(p\right)=\;\left|\underset{q\in R\left(p\right)}{\sum }g_{p,q}\cdot {\left(\partial_{y}S\right)}_{q}\right|.$$

The objective function defined in Equation (A1) is non-convex and can be solved using the linear optimization process with the penalty of quadratic measure proposed by Xu et al. [43]. For a practical implementation, the available Matlab^®^ code by Xu et al. [43] was used with the settings stated in Table A1. These settings were selected by an extensive practical testing where the overall functionality of both HRA and LRA removal algorithms was evaluated and optimized using described synthetic data by a combination of both qualitative and quantitative evaluation (brute-force search with SSIM [37] as a validation metric).

**Table A1:** Relative total variation (RTV) texture extraction settings used within HRA and LRA removal algorithms.

Parameter	HRA Removal	LRA Removal
*Λ*	0.005	0.050
*Ε*	0.020	0.030
*Σ*	6	1

## Appendix B

**Table A2:** Nominal parameters of both used cameras without a lens unit.

Technical Features	CCD Camera	sCMOS Camera
Array size	3320(H) × 2500(V)	2048(H) × 2048(V)
Pixel size	5.4 µm	6.5 µm
Sensor diagonal	22.5 mm	18.8 mm
Nonlinearity	<1%	0.2%
Dynamic range	2300: 1	21,400: 1
Acquisition gain	0.45 e-/ADU	0.52 e-/ADU
Readout noise	11 e-rms	1.4 e-rms
Readout rate	8 Mpix./s (approx. 1 fps)	40 fps (@ 16 bit)
Dark current	0.001 e-/pix./s −35 °C	0.14 e-/pix./s @ 0 °C
Binning	Independent on-chip binning in x, y	2 × 2, 3 × 3, 4 × 4, 8 × 8
Peak quantum efficiency	56% @ 540 nm	82% @ 550 nm
Shutter type	Electromechanical	Rolling shutter
Data interface	USB 2.0	USB 3.0

## Appendix C

### Appendix C.1. Proposed Method—Used Settings

These settings were selected by an extensive practical testing where the overall functionality of the algorithms was evaluated and optimized using synthetic data by a combination of both qualitative and quantitative evaluation (brute-force search with SSIM [37] as a validation metric).

**Table A3:** HRA removal algorithm settings.

Parameter	Value
*L*	10% of sinogram rows
*R*_1_	0.05
*R*_2_	70% of sinogram rows
*R*_3_	0.25% of sinogram columns

**Table A4:** LRA removal algorithm settings.

Parameter	Value
*F*	129
*L*	10% of sinogram rows
*R*_1_	0.02
*R*	6

### Appendix C.2. Other Methods—Used Settings

**Table A5:** List of the other methods and used settings (notations of the parameters are adopted from the original works).

Method	Notation	Settings
Münch [13]	M1	Wavelet: DB7; Decomposition level: 4; Damping factor: 1.3
Eldib [15]	M2	Filter size: 15; Standard deviation: 10
Vo [21]	M3	Algorithms sequence: 6, 5, 3; R = 7; Filter size for algorithms 5 and 6: 81; Filter size for algorithm 3: 31

## References

[1] Yousuf M., Asaduzzaman M. (2009). An Efficient Ring Artifact Reduction Method Based on Projection Data for Micro-CT Images. J. Sci. Res..

[2] Lifton J., Liu T. (2019). Ring artefact reduction via multi-point piecewise linear flat field correction for X-ray computed tomography. Opt. Express.

[3] Seibert J., Dobbins J., Boone J., Boone J., Lindfors K. (1998). Flat-field correction technique for digital detectors. Medical Imaging 1998: Physics of Medical Imaging, Proceedings of the SPIE, San Diego, CA, USA, 21–26 February 1998.

[4] Vågberg W., Larsson J., Hertz H. (2017). Removal of ring artifacts in microtomography by characterization of scintillator variations. Opt. Express.

[5] Boin M., Haibel A. (2006). Compensation of ring artefacts in synchrotron tomographic images. Opt. Express.

[6] Croton L., Ruben G., Morgan K., Paganin D., Kitchen M. (2019). Ring artifact suppression in X-ray computed tomography using a simple, pixel-wise response correction. Opt. Express.

[7] Van Nieuwenhove V., De Beenhouwer J., De Carlo F., Mancini L., Marone F., Sijbers J. (2015). Dynamic intensity normalization using eigen flat fields in X-ray imaging. Opt. Express.

[8] Davis G., Elliott J. (1997). X-ray microtomography scanner using time-delay integration for elimination of ring artefacts in the reconstructed image. Nucl. Instrum. Methods Phys. Res. Sect. A Accel. Spectrom. Detect. Assoc. Equip..

[9] Doran S., Koerkamp K., Bero M., Jenneson P., Morton E., Gilboy W. (2001). A CCD-based optical CT scanner for high-resolution 3D imaging of radiation dose distributions: Equipment specifications, optical simulations and preliminary results. Phys. Med. Biol..

[10] Jenneson P., Gilboy W., Morton E., Gregory P. (2003). An X-ray micro-tomography system optimised for the low-dose study of living organisms. Appl. Radiat. Isot..

[11] Ji D., Qu G., Hu C., Liu B., Jian J., Guo X. (2017). Anisotropic total variation minimization approach in in-line phase-contrast tomography and its application to correction of ring artifacts. Chin. Phys. B.

[12] Raven C. (1998). Numerical removal of ring artifacts in microtomography. Rev. Sci. Instrum..

[13] Münch B., Trtik P., Marone F., Stampanoni M. (2009). Stripe and ring artifact removal with combined wavelet—Fourier filtering. Opt. Express.

[14] Sadi F., Lee S., Hasan M. (2010). Removal of ring artifacts in computed tomographic imaging using iterative center weighted median filter. Comput. Biol. Med..

[15] Eldib M., Hegazy M., Mun Y., Cho M., Cho M., Lee S. (2017). A Ring Artifact Correction Method: Validation by Micro-CT Imaging with Flat-Panel Detectors and a 2D Photon-Counting Detector. Sensors.

[16] Anas E., Lee S., Kamrul Hasan M. (2011). Classification of ring artifacts for their effective removal using type adaptive correction schemes. Comput. Biol. Med..

[17] Anas E., Kim J., Lee S., Hasan M. (2011). High-quality 3D correction of ring and radiant artifacts in flat panel detector-based cone beam volume CT imaging. Phys. Med. Biol..

[18] Rashid S., Lee S., Hasan M. (2012). An improved method for the removal of ring artifacts in high resolution CT imaging. EURASIP J. Adv. Signal Process..

[19] Anas E., Lee S., Hasan M. (2010). Removal of ring artifacts in CT imaging through detection and correction of stripes in the sinogram. Phys. Med. Biol..

[20] Ashrafuzzaman A., Lee S., Hasan M. (2011). A Self-Adaptive Approach for the Detection and Correction of Stripes in the Sinogram: Suppression of Ring Artifacts in CT Imaging. EURASIP J. Adv. Signal Process..

[21] Vo N., Atwood R., Drakopoulos M. (2018). Superior techniques for eliminating ring artifacts in X-ray micro-tomography. Opt. Express.

[22] Kim Y., Baek J., Hwang D. (2014). Ring artifact correction using detector line-ratios in computed tomography. Opt. Express.

[23] Wei Z., Wiebe S., Chapman D. (2013). Ring artifacts removal from synchrotron CT image slices. J. Instrum..

[24] Sijbers J., Postnov A. (2004). Reduction of ring artefacts in high resolution micro-CT reconstructions. Phys. Med. Biol..

[25] Kyriakou Y., Prell D., Kalender W. (2009). Ring artifact correction for high-resolution micro CT. Phys. Med. Biol..

[26] Liang X., Zhang Z., Niu T., Yu S., Wu S., Li Z., Zhang H., Xie Y. (2017). Iterative image-domain ring artifact removal in cone-beam CT. Phys. Med. Biol..

[27] Yan L., Wu T., Zhong S., Zhang Q. (2016). A variation-based ring artifact correction method with sparse constraint for flat-detector CT. Phys. Med. Biol..

[28] Paleo P., Mirone A. (2015). Ring artifacts correction in compressed sensing tomographic reconstruction. J. Synchrotron Radiat..

[29] Titarenko S., Withers P., Yagola A. (2010). An analytical formula for ring artefact suppression in X-ray tomography. Appl. Math. Lett..

[30] Yu J., Imai F., Sampat N., Collins D., Yasan A., Xiao F., Bae S., Ramaswami S. Hot pixel reduction in CMOS image sensor pixels. Proceedings of the SPIE 7537: Digital Photography VI 2010.

[31] Savitzky A., Golay M. (1964). Smoothing and Differentiation of Data by Simplified Least Squares Procedures. Anal. Chem..

[32] Rigaku Corporation: Nano3DX—X-ray Microscope. https://www.rigaku.com/en/products/xrm/nano3dx.

[33] Rigaku Corporation: Compact Two-Dimensional CC Detector. https://www.rigaku.com/en/products/detectors/micron.

[34] Rigaku Corporation: Compact Two-Dimensional sCMOS Detector. https://www.rigaku.com/en/products/detectors/micron-cmos.

[35] Šalplachta J., Zikmund T., Horváth M., Takeda Y., Omote K., Pína L., Kaiser J. CCD and scientific-CMOS detectors for submicron laboratory based X-ray Computed tomography. Proceedings of the 9th Conference on Industrial Computed Tomography (iCT).

[36] Van Aarle W., Palenstijn W., Cant J., Janssens E., Bleichrodt F., Dabravolski A., De Beenhouwer J., Joost Batenburg K., Sijbers J. (2016). Fast and flexible X-ray tomography using the ASTRA toolbox. Opt. Express.

[37] Wang Z., Bovik A., Sheikh H., Simoncelli E. (2004). Image Quality Assessment: From Error Visibility to Structural Similarity. IEEE Trans. Image Process..

[38] Altman D., Bland J. (1994). Statistics Notes: Diagnostic tests 1. BMJ.

[39] Altman D., Bland J. (1994). Statistics Notes: Diagnostic tests 2. BMJ.

[40] Zou K., Warfield S., Bharatha A., Tempany C., Kaus M., Haker S., Wells W., Jolesz F., Kikinis R. (2004). Statistical validation of image segmentation quality based on a spatial overlap index1. Acad. Radiol..

[41] Friedman S., Fung G., Siewerdsen J., Tsui B. (2013). A simple approach to measure computed tomography (CT) modulation transfer function (MTF) and noise-power spectrum (NPS) using the American College of Radiology (ACR) accreditation phantom. Med. Phys..

[42] ASTM E1695-95: Standard Test Method for Measurement of Computed Tomography (CT) System Performance 2013. https://compass.astm.org/Standards/HISTORICAL/E1695-95R13.htm.

[43] Xu L., Yan Q., Xia Y., Jia J. (2012). Structure extraction from texture via relative total variation. ACM Trans. Graph..

